# Toward Precision Medicine with Human Pluripotent Stem Cells for Diabetes

**DOI:** 10.1093/stcltm/szac030

**Published:** 2022-05-28

**Authors:** Bushra Memon, Essam M Abdelalim

**Affiliations:** College of Health and Life Sciences, Hamad Bin Khalifa University (HBKU), Qatar Foundation (QF), Education City, Doha, Qatar; Diabetes Research Center, Qatar Biomedical Research Institute (QBRI), Hamad Bin Khalifa University (HBKU), Qatar Foundation (QF), Doha, Qatar; College of Health and Life Sciences, Hamad Bin Khalifa University (HBKU), Qatar Foundation (QF), Education City, Doha, Qatar; Diabetes Research Center, Qatar Biomedical Research Institute (QBRI), Hamad Bin Khalifa University (HBKU), Qatar Foundation (QF), Doha, Qatar

**Keywords:** hPSCs, insulin-secreting cells, pathogenesis, drug development, personalized therapy

## Abstract

Although genome profiling provides important genetic and phenotypic details for applying precision medicine to diabetes, it is imperative to integrate in vitro human cell models, accurately recapitulating the genetic alterations associated with diabetes. The absence of the appropriate preclinical human models and the unavailability of genetically relevant cells substantially limit the progress in developing personalized treatment for diabetes. Human pluripotent stem cells (hPSCs) provide a scalable source for generating diabetes-relevant cells carrying the genetic signatures of the patients. Remarkably, allogenic hPSC-derived pancreatic progenitors and β cells are being used in clinical trials with promising preliminary results. Autologous hiPSC therapy options exist for those with monogenic and type 2 diabetes; however, encapsulation or immunosuppression must be accompanied with in the case of type 1 diabetes. Furthermore, genome-wide association studies-identified candidate variants can be introduced in hPSCs for deciphering the associated molecular defects. The hPSC-based disease models serve as excellent resources for drug development facilitating personalized treatment. Indeed, hPSC-based diabetes models have successfully provided valuable knowledge by modeling different types of diabetes, which are discussed in this review. Herein, we also evaluate their strengths and shortcomings in dissecting the underlying pathogenic molecular mechanisms and discuss strategies for improving hPSC-based disease modeling investigations.

## Graphical Abstract

**Graphical Abstract:**
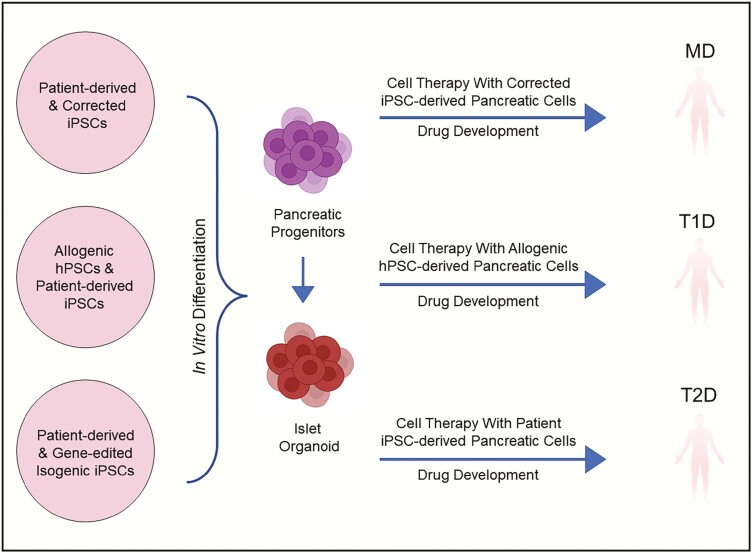

**Significance Statement**

Diabetes is one of the leading causes of morbidity and mortality worldwide. Understanding the mechanisms underlying diabetes pathogenesis has been hindered due to lack of appropriate models that recapitulate human physiology. In vitro human pluripotent stem cells (hPSC) models are promising sources for precision medicine in diabetes. Integrating hPSCs with gene editing and genomics approaches offer a useful tool for more accurate pathogenesis investigation, drug development, and cell therapy. This review summarizes the established hPSC models for different types of diabetes providing valuable knowledge about diabetes pathogenesis in humans and paving the way for personalizing treatment.

## Introduction

Diabetes is a complex, multifactorial disease characterized by increased blood glucose levels (hyperglycemia) due to defects or loss of pancreatic β cells. The most common subtypes of diabetes are type 1 (T1D) and type 2 (T2D).^1^ Type 1 diabetes is characterized by marked loss of pancreatic β cells resulting from autoimmune attack. Type 2 diabetes accounts for 90%-95% of cases worldwide and typically affects older individuals.^1^ Type 2 diabetes involves multiple pathophysiological mechanisms, including insulin resistance in the insulin-target tissues and β-cell dysfunction, which occurs gradually.^2^ Patients with T2D do not usually require insulin at the early stage of the disease; however, many patients require insulin therapy over the disease course.^3^ Monogenic diabetes (MD) is another subtype, which occurs due to mutations/defects in single genes crucial for pancreatic β-cell function.^4^ The types of MD include neonatal diabetes (ND), diagnosed before 6 months of age, and maturity-onset of diabetes of the young (MODY), which is mainly diagnosed in early adulthood.^4^

**Figure 1.**
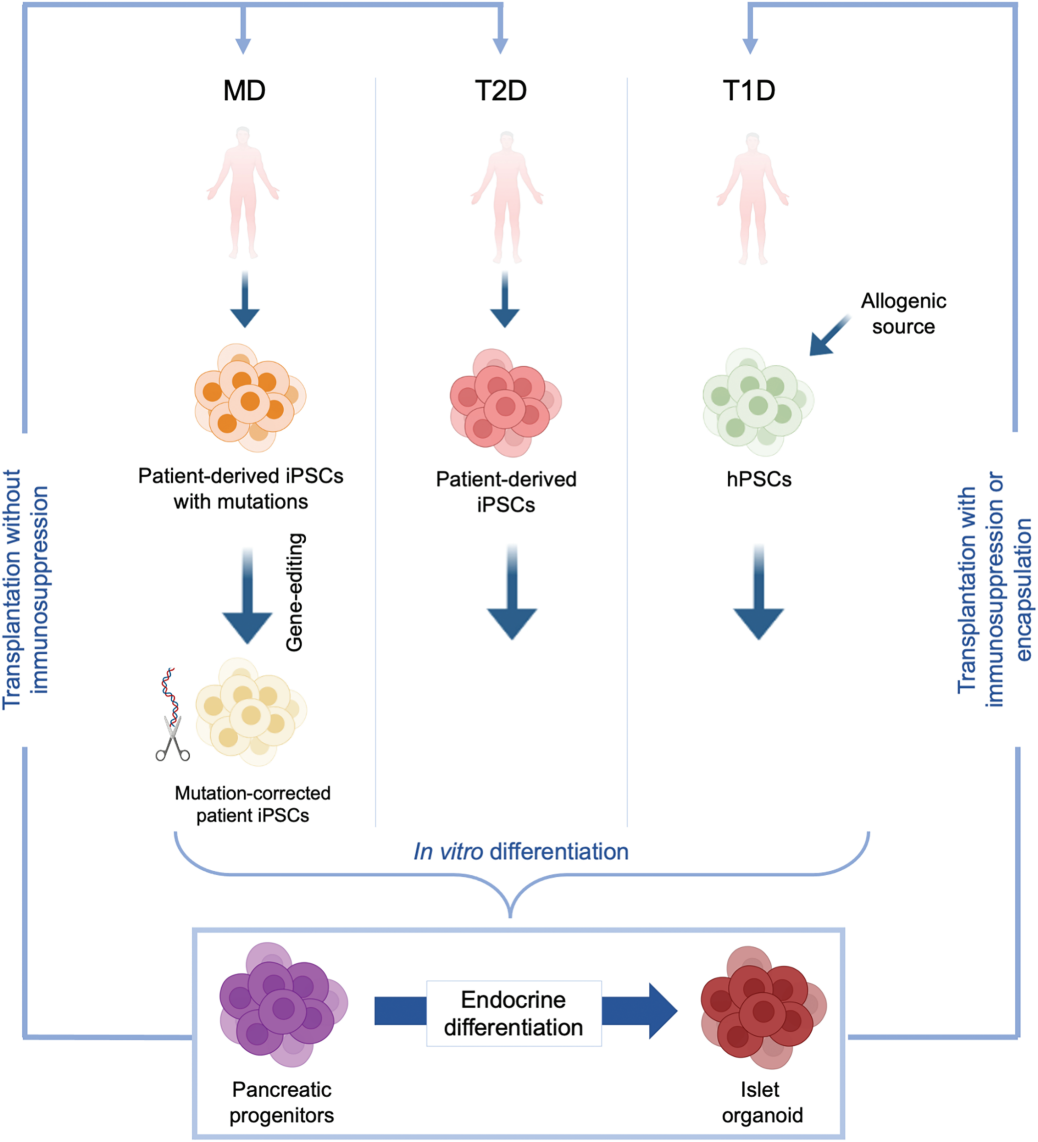
Using patient-derived hPSCs for β-cell replacement therapy and personalized treatment for patients with diabetes. Induced pluripotent stem cells (iPSCs) can be generated from patients with different forms of diabetes. Induced pluripotent stem cells generated from patients with monogenic diabetes (MD) due to specific mutations can be corrected to generate healthy iPSCs. Corrected MD-iPSCs and those generated from patients with type 2 diabetes (T2D) can be differentiated into pancreatic progenitors and then insulin-secreting β cells. The differentiated cells can be used for autologous cell therapy where the cells are transplanted back into T2D and MD patients without the need for immunosuppression. However, Patients with T1D would still require immunosuppression, or the pancreatic cells can be transplanted in a capsule to avoid using immunosuppressants.

The genetic component plays a major role in the pathogenesis of different forms of diabetes. However, until recently it was difficult to study human phenotypes due to lack of the suitable human models recapitulating human pancreatic development. The recent progress in the human pluripotent stem cell (hPSC) technology offers new tools to understand the defective phenotypes associated with each diabetes subtype and allows the development of novel personalized therapies^5^ (Fig. 1).

## Pluripotent Stem Cell Therapy for Diabetes Has Reached Clinical Trials: Opportunities and Challenges

Human pluripotent stem cell can sequentially differentiated to pancreatic β cells that secrete insulin in response to glucose by directing them through intermediary lineages including pancreatic progenitors, using a cocktail of signaling molecules that mimic in vivo human embryonic development.^6^ Pancreatic progenitors expressing the 2 key transcription factors (TF), PDX1 and NKX6.1, give rise to insulin-secreting β cells both in vitro and in vivo.^6^ Co-expression of NKX6.1 in PDX1+ progenitors is crucial for generating monohormonal, functional β cells, while its absence redirects them toward polyhormonal, non-functional or α-cell fate.^7,8^ Of note, clinical trials for treatment of T1D have been initiated using hPSC-derived pancreatic lineages (Table 1). ViaCyte, leading the clinical trials, introduced 2 products; PEC-Encap (NCT02239354), where the implanted hPSC-derived pancreatic progenitors are macro-encapsulated to prevent host’s immune cells from interacting with the implanted ones, whereas in PEC-Direct, the implanted cells are directly in contact with the bloodstream through ports allowing direct engraftment in these regions (NCT03163511). Approximately, an average islet equivalent of only 5300 IEQ/kg body weight or 250-500 million PEC-01 cells was implanted, which is lower than the recommended total of >11 000 IEQ/kg of body weight that allows insulin independence when transplanted into the liver.^9,10^ The first phases of both clinical trials assess the safety and efficacy of the products. Preliminary results have demonstrated that the implanted progenitors differentiate to insulin-expressing cells and have the capacity to respond to glucose. The responders that showed C-peptide secretion had a higher number of insulin-positive cells per graft on average compared with non-responders that do not show C-peptide secretion.^10^ At 3 months post-implant, in contrast to all 6 of the responders, only 4 out of 11 non-responders showed a maximal of stimulated C-peptide secretion above 50 ng/mL.^10^ However, the studies also presented limitations in overcoming hyperglycemia in these patients with T1D in the current setting due to insufficient β-cell mass generated and incomplete engraftment resulting from deposition of extracellular matrix in the ports of the encapsulation device by the infiltrating myofibroblasts.^9,10^ Of note, immunosuppression is required particularly for PEC-Direct as direct contact with the patient’s blood can trigger graft rejection. However, islet transplantation outcomes have described the adverse effects of long-term immune-suppression, which include blood, lymphatic, renal and respiratory disorders, mouth ulcers as well as basal and squamous cell carcinoma (https://citregistry.org/system/files/10th_AR.pdf). Recently, ViaCyte, along with CRISPR Therapeutics, is moving forward with their third product that promises to eliminate the requirement of harmful immunosuppression following transplantation through in another clinical trial. This product comprises of pancreatic progenitors generated from gene-edited hPSCs, interacting directly with the bloodstream that can evade immune system (https://viacyte.com/pipeline/#PEC-QT-VCTX210). Furthermore, Vertex pharmaceuticals, running a Phase I/II clinical trial that transplants fully differentiated hPSC-derived β cells, infused into the hepatic portal vein of hypoglycemic patients with T1D, presented first set of results where they showed a 91% decrease in exogenous insulin administration for their first patient within 90 days, thus providing a huge breakthrough in the field of β-cell replacement therapy (NCT04786262). Vertex only implanted half the target dose and the peak stimulated C-peptide following a mixed meal tolerance test was 560 pmol/L for the patient. In addition, other companies are also endeavoring to develop hPSC-based islets for cell therapy. Sigilon Therapeutics in collaboration with Eli Lilly has their product SIG-002 in the pipeline for clinical trial (https://sigilon.com/eli-lilly-inks-63m-deal-with-sigilon-to-develop-encapsulated-cell-therapies-for-diabetes/). Another company, Seraxis, has plans to employ their hPSC-derived insulin-secreting cells along with encapsulation system SeraGraft, for T1D clinical trial (https://www.seraxis.com/seraxis-technology/). An important area of debate is the site of islet transplantation. Direct infusion of islets or hPSC-derived β cells into the hepatic portal vein is widely used for clinical use as it has been shown to allow insulin independence; and has several benefits like proximity to insulin metabolization site, ie, liver and a well-established, non-invasive technique. However, objects infused in the hepatic portal vein are non-retrievable, therefore making the method unsuitable for implanting encapsulation devices or pouches. On the other hand, subcutaneous transplantation is safer as it allows graft retrieval; however, evidence hints at a decreased vascularization potential at the site, attributed to the increased risk of fibrosis around the encapsulation device.^11–13^

**Table 1.** Ongoing clinical trials using pancreatic cells derived from hPSCs to treat patients with diabetes.

Clinical trial identifier	Company	Intervention/treatment	Route of administration	Results obtained	Recruitment status
NCT02239354	ViaCyte	hPSC-derived pancreatic progenitors	Subcutaneous	Generates insulin secreting cells 6 months post implantation,	Recruiting by invitation
NCT03163511	ViaCyte	hPSC-derived pancreatic progenitors	Subcutaneous	Generates insulin secreting cells 6 months post implantation with better engraftment	Recruiting
NCT04786262	Vertex	hPSC-derived pancreatic β cells	Hepatic portal vein	Up to 91% reduction in insulin administration within 90 days, at half target dose of implanted cells	Recruiting

**Figure 2.**
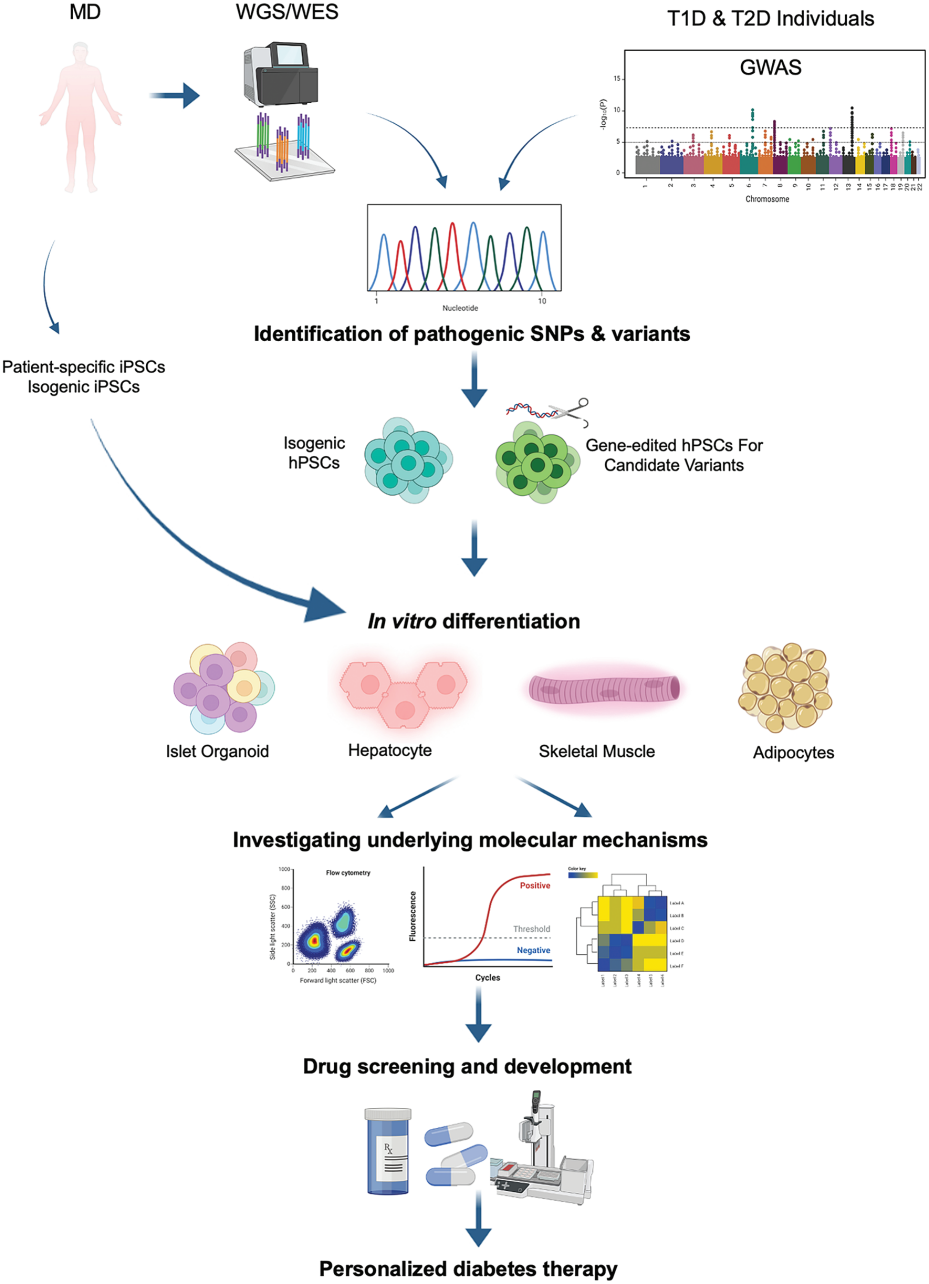
Using hPSCs for personalized therapy for diabetes. Patients with MD are recruited to perform whole-genome sequencing to identify the disease-causing SNPs and variants. Additionally, genome-wide association studies (GWAS) data on T1D- and T2D-associated risk loci can be analyzed to identify candidate variants. hPSCs can be gene-edited to carry the disease-causing variant and, along with its isogenic controls, be differentiated to the target lineage. Likewise, MD patient-derived iPSCs can be edited to correct the mutation and further differentiated to perform functional studies. The differentiated cells are investigated to dissect underlying mechanisms and identify dysregulated pathways. These defective pathways can be reversed using specific molecules or drug libraries to develop candidate-specific personalized therapy.

**Figure 3.**
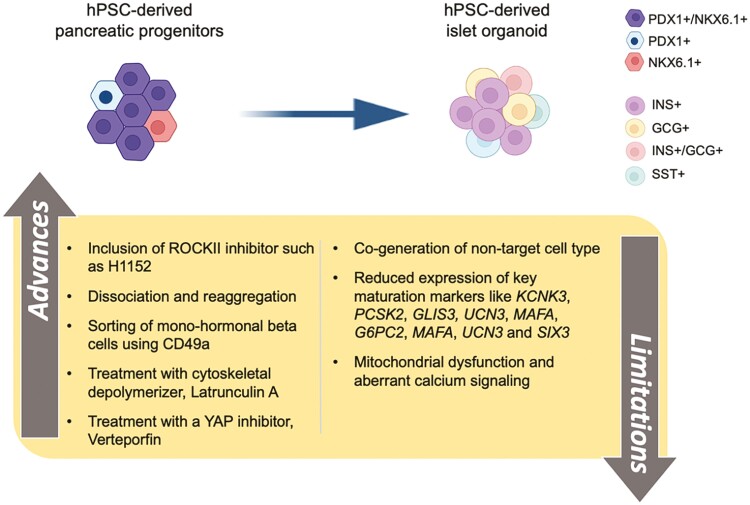
Advances and limitations in generating pancreatic β cells from hPSCs. Multiple modifications in β-cell differentiation protocols are listed, along with limitations in obtaining hPSC-derived β cells representing adult human β cells persist. Use of ROCKII and YAP inhibition, as well as cytoskeletal depolymerizer during endocrine commitment improves proportion and functionality of the generated β cells. Furthermore, physical manipulation such as disassociation and reaggregation of the hPSC-derived endocrine clusters also improves maturity of β cells and eliminates non-β cells, such as α cells, delta cells or non-committed progenitors and polyhormonal cells, that are normally present in differentiated endocrine clusters along with β cells. However, key maturation markers are still absent from the differentiated β cells along with aberrant calcium signaling and dysfunctional mitochondria.

Nonetheless, hPSC-derived β cells are more quick to exert control over glycemic levels upon transplantation compared with hPSC-derived pancreatic progenitors.^6^ Human pluripotent stem cell-derived β cells can also provide valuable models recapitulating the disease phenotype caused by mutations, risk alleles, and variants in vitro. Undertsanding the mechanisms underlying these diseases will allow development of therapeutic options for treatment of specific types of diabetes (Fig. 2). In vitro differentiation protocols have advanced in obtaining β cells; however, several impediments remain. Human pluripotent stem cell-derived β cell differentiation protocols have been improved by employing efficiency modulators such as Rock II^14^ and YAP^15^ inhibitors as well as cytoskeletal depolymerizer,^16^ in addition to identification of a specific surface marker for functional β cells such as CD49a^17^ that allows their purification from other co-generated endocrine and progenitor cells. Challenges encompassing obtaining an identical adult human β-cell transcriptional and functional profile for hPSC-derived β cells are yet to be fully solved. The advances and hurdles in obtaining hPSC-derived functional β cells are summarized in Fig. 3.

## In Vitro Modeling of MD

Genes associated with MD are not only expressed in pancreatic islet cells but also mutations in TFs that govern pancreatic development, such as *PDX1*, *NGN3*, *PTF1A*, *NEUROD1*, *RFX6*, *NKX2.2*, *MNX1*, *GLIS3*, *GATA4*, *GATA6*, *HNF1B* lead to permanent ND (PNDM).^5^ Homozygous mutations in *GATA4*, *GATA6*, *PDX1*, and *PTF1A* cause agenesis of the pancreas, whereas mutations in *WSF1*, *EIF2AK3*, *IER3IP*, and *FOXP* lead to syndromic MD.^5^ Furthermore, mutations in proteins related to glucose metabolism including *GCK* (*Glucokinase*) and *GLUT2* (*SLC2A2*) also lead to PNDM. Maturity-onset of diabetes of the young is associated with defects in 14 different genes, the most common ones being *GCK*, *HNF4A*, *HNF1A*, and *HNF1B*.^5^

The cellular and molecular bases of MD remain largely unknown due to lack of the models that can recapitulate the genotype-phenotype correlations. Several of these genetic mutations show contradicting pathophysiology in mouse models compared with humans. Therefore, hPSCs have shown great promise in recapitulating the correct phenotype for the pathogenic human genotype. Induced PSCs (iPSCs) have been established from patients with MD carrying mutations or those mutations have been introduced into normal hPSC lines using gene-editing tools. Patient-specific iPSCs and/or gene-edited hPSCs have been recently used to investigate the mechanisms and biological processes altered by those genetic defects during the differentiation of pancreatic β cells and/or insulin-target cells (Fig. 2).

Mutations in the *INSULIN* (*INS)* gene are one of the leading causes of PNDM; however, only 2 groups have attempted to model it using iPSCs.^18,19^ While both models highlighted insulin deficiency, the underlying mechanisms differ in each case. Heterozygous mutation in protein-coding region of the *INS* gene responsible for formation of the proinsulin inter-chain disulfide bonds showed reduced proliferation along with an increase in ER stress resulting from the misfolding and accumulation of the proinsulin molecule, downregulated mTORC signaling with the rate of apoptosis being unaffected.^18^ On the other hand, PNDM iPSC model due to defect in the translational start site (codon 1) of *INS* gene (*INS*^*ATG>ATA*^), showed that INS-negative PDX1+/NKX6.1+/MAFA+ β cells do form during β-cell differentiation of the mutant *INS*^*ATG>ATA*^ iPSCs, indicating that loss of INSULIN does not inhibit the development of mature β cells.^19^

Induced PSC models for mutations in PDX1, the master regulator of pancreatic development, elucidate how the nature of mutation and its effect on functionality of the encoded protein affects pancreatic differentiation, for the same gene. Single allelic iPSC deletion models of PDX1, *PDX 1*^*L36fs/+*^ hESCs, and *PDX1*^*A34fs/+*^ hESCs with reduced PDX1 protein, showed a decreased expression of endocrine markers such as INS, GLUCAGON (GCG), and SOMATOSTATIN (SST),^20^ in contrast to rodents where heterozygous deletion of Pdx1 does not cause endocrine developmental abnormalities.^21,22^ On the other hand, *PDX1*^*P33T/P33T*^ and *PDX1*^*C18R/C18R*^ mutations in the transactivation domain of PDX1 showed that co-expression of PDX1 and NKX6.1 at the progenitor stage is only impacted by the *PDX1*^*+/−*^ and *PDX1*^*P33T/P33T*^ but not *PDX1*^*C18R/C18*^.^23^ Expression of *PDX1*, *NKX6.1*, *MAFA*, *PAX6*, *SLC30A8*, and *KCNJ11* is less in the *PDX1*^*+/−*^ and *PDX1*^*P33T/P33T*^ but not in *PDX1*^*C18R/C18R*^ β cells, indicating a differential effect on PDX1’s ability to bind its interacting partners and regulate target gene expression by various point mutations in the transactivation domain of PDX1.^23^ Overall, in comparison to controls, the PDX1 mutant cell lines showed a hindered development of pancreatic progenitors as well as endocrine cells and had a defective β-cell functional response.^23^

Pancreatic agenesis can be caused by heterozygous mutations in *GATA6* gene^24,25^; however, only one study showed that heterozygous loss-of-function GATA6 mutation decreases endodermal efficiency modestly.^26^ Interestingly, *GATA6* haploinsufficiency it does not lead to any pancreatic and non-pancreatic abnormalities in mice.^27^ On the other hand, *GATA6*^*−*/*−*^ patient iPSCs could not give rise to endoderm.^26,28,29^ Furthermore, retinoic acid (RA) that is widely added in in vitro pancreatic differentiation protocols, could mask the effect of *GATA6* haploinsufficiency on pancreatic development, which may lead to a discrepancy in results obtained for models with similar genetic makeup by differentiation protocols using varying cytokine concentrations. Indeed, excluding RA from in vitro differentiation abolished pancreatic gene expression in *GATA6*^*−*/*−*^ iPSCs.^28^ Furthermore, studies on the established *GATA6*^*−*/*−*^ models consistently indicated a defect in endocrine cell differentiation and reduced number of INS^+^ cells^26,28,29^; however, functional response of β cells to elevated glucose is impacted differentially in these GATA6 loss-of-function models. Interestingly, GATA6 heterozygous mutations have been shown to have a varying degree of penetrance, indicating that additional factors may complement GATA6 functions during development. The mutant *GATA6*^*−*/*−*^ null iPSCs had decreased levels of GATA4 in the endodermal cells generated from them, wherein, the hindered endodermal gene expression in *GATA6*^*−*/*−*^ iPSCs was rescued by the overexpression of *GATA4*.^28^ Another group generated *GATA6*^−/+^*GATA4*^−/+^ hPSCs and demonstrated that heterozygous loss of *GATA6* when further potentiated by decreased *GATA4* dosage leads to downregulated pancreatic differentiation.^29^ The study further validated the fine tuning of pancreatic development by GATA6/GATA4 gene dosage.^29^ Another study investigated how the presence of SNP in non-coding region of *GATA6* may cause phenotypic variation in patients.^30^ Patients that carried an SNP (G>A) in the 3ʹ of GATA6 along with a heterozygous mutation in GATA6 suffered from pancreatic agenesis compared with those who did not carry this accompanying SNP.^30^*GATA6*^+/*−*^ iPSCs derived from patients with pancreas agenesis showed a decreased amount of GATA6 protein and a downregulated pancreatic program despite of mutation correction compared with control hPSC cells.^30^ hPSCs introduced with the 3ʹ SNP and heterozygous loss of function mutation in the GATA6 alleles, showed the least numbers of NKX6.1+ cells and highest number of SOX2+ (stomach lineage) in the Mel^+/mut | A/A^ background compared to Mel^+/+ | G/G^, Mel^+/mut | G/G^ and Mel^+/+ | A/A^.^30^ The genome-edited iPSCs helped determine that the region carrying this 3ʹ SNP comprises of a binding site for TF RORa that regulates GATA6 expression, which is compromised in the presence of the SNP and a compounding *GATA6*^+/*−*^ mutations redirects the foregut progenitors to an alternative fate leading to deformation of the pancreas.^30^ These findings altogether highlight the importance of using hPSC models in understanding how different factors such as external signaling, presence of accompanying SNPs in non-coding regions and gene dosage of TFs affect disease phenotype. This aids in development of genotype-specific personalized diabetes treatment.

Induced PSCs models to recapitulate the effect of *HNF4A* inactivating mutations (MODY1) have been generated but with contradicting results which do not completely explain the underlying causes.^31–33^ Patient-derived iPSCs carrying a truncating *HNF4A*^p.Ile271fs/+^ mutation, which sequesters HNF4A in the cytoplasm, showed a hindered foregut endodermal development, with downregulation of *HHEX*, *PDX1*, *GATA4* among others and a diminished expression of its target gene, *HNF1a*, in *HNF4A*^p.Ile271fs/+^-derived pancreatic β cells.^32^ Complementing this, endocrine cells generated from MODY1-iPSCs carrying the truncating p.Ile271fs mutation expressed some key β-cell markers; however, both studies did not generate glucose-responsive β cells.^31^ Conversely, another model for MODY1, Q268X substitution, showed a modest increase in pancreatic endocrine gene expression in the progenitor stage which may be due to compensatory mechanisms by the pancreatic cells to surpass the loss of HNF4A levels. The studies, overall, did not use efficient protocols for mature, functional β-cell generation and hence failed to assess the disease-relevant cells, in addition to deciphering the impact on pancreatic endocrine development due to *HNF4A* mutations more thoroughly. Furthermore, gene correction of the mutations to generate isogenic control cell lines would enhance gauging of abnormalities that contribute to MODY1 phenotype more robustly.

Heterozygous mutations in *HNF1B* leads to MODY5 associated with pancreatic hypoplasia^34,35^; however, the causative mechanisms are not fully understood. *HNF1B*^*S148L/+*^ (S148L mutation affects the DNA binding ability of HNF1B) iPSC-derived pancreatic lineages showed a compensatory upregulation of multiple pancreatic TFs, such as *PDX1*, *FOXA2*, *TCF2*, *ISL1*, *MNX1*, *RFX6*, *GATA4*, and *GATA6* in comparison to those generated from Ctr-iPSCs; however, a decrease in *PAX6*. The decrease in PAX6 may explain the presence of diabetes, although HNF1B does not bind *PAX6* directly.^36^ Another model for *HNF1B*^*−/+*^ showed an impaired β cell differentiation and reduced proliferation of the foregut and pancreatic progenitors, which addressed the underlying cause for pancreatic hypoplasia.^37^ The *HNF1B*^*−/+*^ pancreatic progenitors, compared to their isogenic controls, also showed decreased expression of *HNF1A* and the Hippo signaling regulator, *ROBO2*, the downstream targets of HNF1B.^37^

Heterozygous mutations in *HNF6* cause juvenile-to-adult-onset diabetes characterized by defective insulin secretion, whereas complete loss of HNF6 leads to syndromic diabetes.^38^ Pancreatic progenitors generated from *ONECUT1*^*−/−*^ and the 2 disease-causing variants, ONECUT1-p.E231X and ONECUT1-p.E231D, hPSCs showed a decreased expression of the key β-cell marker NKX6.1, along with NKX6.2 and NKX2.2, due to decreased occupancy of their enhancers by ONECUT1 leading to a reduction in their activity. Thus, the hPSC model for HNF6 deficiency helped to understand that the resultant diabetes is caused by diminished interaction of the ONECUT1 C terminus with NKX6.1/NKX2.2 and a reduced chromatin accessibility during transition from posterior foregut to pancreatic progenitors which in turn regulates development of adequate mass.

Mutations in another HNF family member, *HNF1A*, are responsible for the majority of MODY cases.^39^ human embryonic stem cells (hESCs) containing *HNF1A*^*+/−*^ and *HNF1A*^*−/−*^ mutations when differentiated to pancreatic endocrine cells showed that balance between expression levels of different hormones was lost. *HNF1A* dose reduction led to an increase in GCG and GHRELIN whereas a striking decrease in INS levels was observed.^40^*HNF1A*^*+/−*^ and *HNF1A*^*−/−*^ displayed abolished functional response and an impaired cellular respiration capacity.^40^ The study identified a lncRNA, *LINC01139* or *LINKA*, as a target of HNF1A, which promotes α-cell differentiation.^40^ These studies together revealed the potential of iPSC technology in dissecting human disease mechanisms; however, factors such as efficiency of differentiation protocols or presence of relevant cell type in culture may significantly affect the impact of genotype on the observed phenotype in vitro.

Another key TF that causes MD is NEUROG3, which is the master regulator of endocrine pancreas development. In mouse, loss of *Neurogenin3* leads to a complete lack of β cells.^41^ Interestingly, patients carrying homozygous mutations in the *NEUROG3* gene exhibit detectable blood C-PEPTIDE levels, indicating the presence of a few functional β cells in the pancreas.^42–44^ This is because a minimum of 10% of NEUROG3 expression is sufficient to induce generation of β cells in humans.^45^ On the other hand, mice with *Neurogenin3* haploinsufficiency develop decreased islet mass and are diabetic,^46^ whereas patients carrying *NEUROG3* heterozygous mutations have normal glucose tolerance.^43^ hESCs lacking *NEUROG3* can generate a small number of pancreatic β cells (~0.5% C-PEP^+^ cells).^20^ Furthermore, differentiation of hESC lines carrying *NEUROG3*^*R107S/R107S*^ mutation showed a dramatic decrease in the number of INS^+^ cells at the β cell stage.^20^

Homozygous *RFX6* mutations cause Mitchell-Riley syndrome associated with PNDM.^47^ RFX6 plays a crucial role during pancreatic development in regulating the differentiation of pancreatic progenitors into endocrine cells^48,49^ and in maintaining β-cell function.^50,51^ In line with this, *RFX6*^−/−^ hESCs generated a reduced population of pancreatic progenitors (~ 40% decrease in the PDX1+ cells) and lacked INS^+^ and GCG^+^ endocrine cells.^20^ Therefore, *RFX6* mutations cause PNDM due to aberrant development of the pancreatic progenitors and endocrine lineages. Patient-specific hapoloinsufficiency model for FOXA2 showed severe pancreatic phenotypes, with significant reduction in pancreatic progenitors and endocrine lineages.^52^

The efficiency of in vitro pancreatic differentiation protocols also affects the gene expression of key MD-associated genes as well as fate specification of the disease-relevant pancreatic lineages. One key example is the induced expression of *GLIS3* during pancreatic differentiation using more efficient and recent protocols that generate monohormonal β cells^53,54^ compared to the previous ones.^55,56^*GLIS3*^*−/−*^ hESCs when differentiated using inefficient protocol showed no significant differences compared to control hESCs^20,54^; however, showed robust impact on β cell functionality and apoptosis using more enhanced protocol.^54^ Notably, *GLIS3*^*−/−*^ hESCs have been used to discover galunisertib, a TGFβ inhibitor, for rescuing from apoptosis associated with reduction of GLIS3 levels in β cells.^54^ The results of the latter study are consistent with evidence from mice models that GLIS3 regulates pancreatic β cell development and insulin secretion.^53^

Induced PSC models for *YIPF5* and *WFS1* mutations show ER stress as the main pathogenic mechanism in hPSC-derived β cells. *YIPF5* (YIP1 domain family member 5) that leads to ND and microcephaly as well as *WFS1* that causes Wolfram Syndrome affect pancreatic β-cell function. Induced PSCs derived from patients carrying the *YIPF5*^Ile98Ser^ mutation did not affect insulin content and glucose-stimulated insulin secretion (GSIS); however, showed increased ER stress in the differentiated β cells and accumulation of proinsulin.^57^ β cells derived from iPSCs carrying the homozygous *WFS1* mutation, compared with their corrected isogenic cells, showed reduced expression of INS, GCG, a diminished GSIS, and an increase in SST along with elevated levels of exocrine markers indicating misspecification of mutant β cells during development.^58,59^ Mutant WFS1-iPSCs also showed increased unfolded protein response, which is reversed by treatment with a chemical chaperone 4-phenyl butyric acid indicating that ER stress-related symptoms in pancreatic β cells could be managed using chemical chaperones.^58^ Therefore, iPSC-derived disease models facilitate drug discovery for treatment of diabetes.

Induced PSCs carrying the activating *STAT3* (K392R) mutation showed premature or early induction of endocrine lineage resulting in a smaller pancreas.^60^ NEUROG3 expression is upregulated during the pancreatic progenitor stage, promoting the cells toward INS+/GCG+ polyhormonal and GCG+α cells in the STAT3^K392R^ iPSC-derived pancreatic lineages.^60^ While a previous study established a link between high activity of STAT3 and upregulation of NEUROG3, the authors showed that this increase is not due to augmented DNA binding ability of STAT3 to the NEUROG3 promoter, but rather due increased nuclear translocation of STAT3 due to its enhanced interaction with the nuclear pore proteins.^60^ Importantly, this enhanced premature endocrine induction leads to hypoplasia of the pancreas.

Induced PSCs from multiple other patients with MD have been generated, providing more opportunities for investigating the disease mechanisms through in vitro differentiation and functional assays. The MODY-iPSC models that are yet to be tapped into include MODY1 (*HNF4A*^p.Ile271fs^), MODY2 (*GCK*^V62A^ and *GCK*^p.L146P^), MODY3 (*HNF1A*^P291fsinsC^ and *HNF1A*^p.S142F^), MODY5 (*HNF1B*^R177X^ and *HNF1B*^S148L, and g.1*−*1671del^), MODY8 (*CEL*^C563fsX673^), and MODY13 (*KCNJ11*^p.Glu227Lys^).^5^ Nevertheless, the insights obtained from the MD hPSC models have provided immense knowledge about underlying pathogenic mechanisms. Further studies incorporating genome editing tools and sequencing approach will widen our understanding and help discover novel treatments for diabetes.

## HPSCs to Investigate and Reverse the Underlying Pathogenic Mechanisms in T1D and T2D

Several genome-wide association studies (GWAS) studies have highlighted the role of SNPs in causing T1D and T2D. In case of T1D, the majority of the genetic loci correlated with T1D are associated to the immune system.^61,62^ However, several studies reported that β cells of patients with T1D possess genetic defects making them susceptible to immune attack,^62,63^ indicating that the underlying genetic mechanisms are still unclear. hiPSCs from Patients with T1D have been generated and used to produce β cells carrying T1D genetic signature. Coculture of ER stressed-β cells derived from T1D-iPSCs and control iPSCs with autologous peripheral blood mononuclear cells (PBMCs) revealed that the intensity of T-cell activation is similar between T1D-β cells and control-β cells.^64^ Additionally, T cells responded only to iPSC-derived β cells and not iPSC-derived α cells, indicating that genetically engineered iPSCs to generate β cells that can evade the immune system is a promising option for transplantation therapy.^64^ Furthermore, another key finding is that the immune response is triggered particularly by presence of induced ER stress in these β cells prior to coculture; however, not with IFNγ treatment. These highlight that ER stress in β cells is a trigger for immune response in vitro and therefore may play a role in T1D pathogenesis *in vivo*, consistent with a previous report.^65^ Interestingly, multiple studies have demonstrated the ability of β cells derived from T1D-hiPSCs to secrete INS in response to glucose, similar to healthy iPSCs.^64,66,67^ In contrast, some groups reported that T1D-iPSCs do not generate PDX1^+^ pancreatic progenitors compared with those from non-diabetic healthy controls,^68,69^ and treating with demethylation reagent enhances the generation of PDX1^+^ pancreatic progenitors and subsequently increases the proportion of functional β cells generated suggesting that an epigenetic component may hinder β-cell development in T1D patient-derived iPSCs.^69^ Altogether, these studies are crucial in shedding light on the molecular and cellular pathways underlying T1D and serve as a platform for therapeutic research. Also, iPSCs derived from patients with T1D can be used for generating the immune-effector cells. To gauge a better understanding of T1D pathogenesis, the genetic abnormalities inherent in these T1D-iPSC-derived immune cells can be investigated. Additionally, T cells generated from patient-iPSCs could overcome the shortage of autologous PBMCs in extensive coculture studies, while retaining the patient genetic signature. Also, the generated iPSC-derived immune cells may help in recapitulating the key cell-cell interactions underlying T1D pathogenesis. For example, macrophages have been recently generated from T1D-iPSCs and were able to activate autologous islet-infiltrating T cells upon coculture.^70^ Furthermore, protocols for generation of other key immune effectors like iPSC-derived thymic epithelial cells that can educate developing T cells, are also being optimized to facilitate studies on immune-associated disorders.^71^

Furthermore, T2D GWAS has identified SNPs that have been reported in the MD genes, such as *HNF4A*, *WFS1*, and *HNF1A*.^5^ Additionally, T2D-associated variants have also been mapped to binding sites in DNA regulatory sites of the TFs associated with MD, including HNF1A, HNF4A, HNF1B, PDX1, FOXA2, and NEUROD1.^72^ GWAS-identified candidate genes that are associated with T2D can be genetically modulated in hPSCs to serve as a model, along with its isogenic lines, for investigating their exact roles in T2D pathogenesis. Some of these iPSC models have been generated including those for *CDKAL1*, *KCNQ1*, *SLC30A8*, *KCNJ11*, and *SIX2*.^5^

*SLC30A8*, also known as *ZNT8*, is found on insulin granules in pancreatic β cells. Two of its loss of function, T2D-protective alleles, p.Arg138* and p.Lys34Serfs*50,^73,74^ when introduced in hPSCs (heterozygous for *SLC30A8* p.Arg138* allele and homozygous for *SLC30A8* p.Lys34Serfs*50), and differentiated to β cells showed an improvement in GSIS and higher C-PEPTIDE to PROINSULIN ratio.^75^ Pancreatic β cells derived from hESCs lacking *CDKAL1*, *KCNQ1*, and *KCNJ11* showed stunted insulin secretion and glucose homeostasis.^76^*CDKAL1*^*−/−*^ β cells showed a downregulated expression of the metallothionein family members, which are also associated with diabetes; and overexpression of *MT1E* is able to rescue the β cells from glucolipotoxicity due to loss of CDKAL1.^77^ Furthermore, the hPSC KO model facilitated high-throughput drug screening that identified a FOS/JUN pathway inhibitor, as a factor to overcome some of the *CDKAL1* mutation-associated defects.^76^ Another key hPSC model to study the molecular mechanisms in T2D pathogenesis is the *SIX2*^*−*/*−*^ KO hPSC model.^78^ Lack or deficiency of SIX2 in hPSC-derived mature β cells impairs GSIS and decreases expression of the β-cell maturation markers.^78^ These findings indicate that hPSC models for T2D candidate genes can expand our understanding of the underlying mechanisms that confer susceptibility to T2D development in humans.

Nevertheless, iPSCs derived from insulin resistant^79^ and T2D individuals can be differentiated to β cells or other insulin-target cells and evaluated by bulk and single-cell sequencing in the presence of a diabetic microenvironment in vitro to identify novel pathways and processes affected in T2D. Indeed, several iPSC lines have been established from patients with T2D thus providing the platform to identify genetic regulators beyond GWAS studies.^80,81^ However, since T2D is a polygenic disease, patient with T2D-derived iPSCs models can be used to narrow down the key pathogenic gene networks that cause T2D (Fig. 2).

It is noteworthy to highlight that the use of iPSC models is not limited to studying abnormalities associated with pancreatic β cells in diabetes, but can be expanded to other key cell-types that play a considerable role in diabetes-associated complications. For example, hPSC-derived endothelial cells (ECs) can be used to study genetic dysregulations causing cardiovascular complications. Induced PSC-derived ECs generated from patients with T2D show restricted angiogenic potential, and produce higher amounts of the vasoconstrictor, Endothelin-1.^82^ Endothelial cells generated from iPSCs carrying mutation in MODY3 gene, *HNF1A*, showed a dose-dependent increase in vascular permeability to proinflammatory cytokines, which may contribute to endothelial dysfunction in some MODY3 patients due to a compromised barrier resulting in vascular leakage.^83^

## Conclusion and Future Perspectives

The hPSC-based models that recapitulate disease phenotype in MD has shown great potential and provided valuable insights into deciphering the underlying mechanisms. However, hPSC models should adopt different strategies based on the genotype. Although some GWAS-identified variants have been modeled using hPSCs, these studies do not completely represent the polygenic makeup of T2D and T1D, which result from combined influences of several common variants. Therefore, it is crucial to look beyond hPSC models of rare mutations. To identify the contribution of common variation in the development of polygenic form of diabetes, it may be useful to capture cellular phenotypes associated with the disease in large numbers of iPSC lines generated from patients with polygenic diabetes. These phenotypes cannot be easily anticipated; therefore, it will be useful to use several technical approaches capable of assessing many various cellular characteristics, such as single-cell RNA-sequencing (scRNA-seq) and different genomics profiling tools. Nevertheless, to detect small differences caused by several variants and to minimize the iPSC line-to-line variability and genetic heterogeneity, the experiments should be carefully designed. The obtained molecular and cellular phenotypes could be cross-referenced with other genetic findings generated from large patient cohorts to identify essential diabetes-specific dysregulated pathways and to group patients into well-defined therapy cohorts. Furthermore, vast understanding of disease pathogenesis can be drawn from assessment of epigenetic changes associated with mutations or variants. This can be achieved by analyzing DNA methylation and other histone modification profiles, amongst others, across diseased- and control-iPSCs. However, iPSC models are limited in recapitulating the epigenetic changes acquired due to extrinsic factors, which are one of the primary causes of T2D, as the reprogramming process of iPSCs wipes out epigenetic memory.

Limitations of current in vitro β-cell differentiation protocols may also hinder underpinning of accurate disease mechanisms in mature β cells. Inadequate efficiency and functionality of the β cells generated may not allow discovering of molecular basis for MD or polygenic diabetes caused by defects in genes affecting their functionality. It is likely that if this point is addressed, the discrepancies obtained in studies on similar genotypes by different groups could be minimized resulting in accurate genotype-phenotype correlations.

Importantly, the hPSC therapy for diabetes has reached the clinical trials using pancreatic progenitors and functional β cells. This progress will pave the way toward cell therapy for diabetes and personalized treatment.

## Data Availability

Data sharing is not applicable to this article as no datasets were generated.
